# Genetic variants of Complement factor H gene are not associated with premature coronary heart disease: a family-based study in the Irish population

**DOI:** 10.1186/1471-2350-8-62

**Published:** 2007-09-18

**Authors:** Weihua Meng, Anne Hughes, Chris C Patterson, Christine Belton, Muhammad S Kamaruddin, Paul G Horan, Frank Kee, Pascal P McKeown

**Affiliations:** 1 Centre for Clinical and Population Sciences, Queen's University Belfast, Institute of Clinical Science, Grosvenor Road, Belfast, BT12 6BJ, Northern Ireland, UK; 2 Regional Medical Cardiology Centre, Royal Victoria Hospital, Grosvenor Road, Belfast, BT12 6BA, Northern Ireland, UK; 3 Department of Medicine, Queen's University Belfast, Institute of Clinical Science, Grosvenor Road, Belfast, BT12 6BJ, Northern Ireland, UK

## Abstract

**Background:**

The complement factor H (CFH) gene has been recently confirmed to play an essential role in the development of age-related macular degeneration (AMD). There are conflicting reports of its role in coronary heart disease. This study was designed to investigate if, using a family-based approach, there was an association between genetic variants of the CFH gene and risk of early-onset coronary heart disease.

**Methods:**

We evaluated 6 SNPs and 5 common haplotypes in the CFH gene amongst 1494 individuals in 580 Irish families with at least one member prematurely affected with coronary heart disease. Genotypes were determined by multiplex SNaPshot technology.

**Results:**

Using the TDT/S-TDT test, we did not find an association between any of the individual SNPs or any of the 5 haplotypes and early-onset coronary heart disease.

**Conclusion:**

In this family-based study, we found no association between the CFH gene and early-onset coronary heart disease.

## Background

Coronary heart disease (CHD) remains a leading cause of death worldwide. It is a complex disorder stemming from interactions between multiple genetic and environmental risk factors. There is increasing evidence that one of the key factors generating and maintaining inflammation in the arterial intima is the complement system [1]. Recently, the amount of complement factor H (CFH) was found to be much greater in the superficial layer of human coronary atherosclerotic lesions than in the deeper layers, indicating a potential role of CFH in atherogenesis [2].

CFH is encoded by a single gene (CFH) on human chromosome 1q32. Polymorphisms in this gene have been confirmed to be strongly associated with age-related macular degeneration (AMD) [3-7]. It is suggested that the variants in this gene influence the levels of CFH expression. For example, the Y402H (rs1061170) polymorphism, which is located within binding sites for heparin and C-reactive protein (CRP) [8], was found to change the binding properties and have functional implications [9], whilst the I62V (rs800292) polymorphism is located in exon 2, which contains a C3b binding site [6]. These alterations may lead to complement related damage to blood vessels [4]. CFH variants with more substantial effects may be implicated in the earlier age-of-onset of certain diseases [6]. In 2006, Kardys and colleagues investigated the role of the Y402H polymorphism in the CFH gene and reported an increased risk for myocardial infarction in those who were homozygous for the His variant [10]. However, three subsequent studies have not been able to replicate this result [11-13].

In our study, we investigated the role of CFH gene polymorphisms as risk markers for early-onset CHD in a well-characterised Irish family-based study.

## Methods

### Study population

The entry criteria used in this study have been described elsewhere [14]. Between August 1999 and October 2004 we recruited 1494 individuals from 580 families. All subjects were Caucasian whose four grandparents were born in Ireland. Each family had at least one member affected with early-onset CHD (disease onset ≤55 years for males and ≤60 years for females) and at least one unaffected sibling and/or both parents surviving.

The affected individuals were recruited from the cardiology units of the Royal Victoria Hospital and Belfast City Hospital, Northern Ireland. CHD was defined as the presence of one or more of the following features: (1) a history of acute MI (as defined by WHO criteria); (2) a history of unstable angina (typical chest pain with dynamic ECG changes or minor elevations in cardiac markers); (3) coronary artery disease angiographically (≥ 70% luminal stenosis).

Unaffected siblings were required to: (1) be older than the affected sibling was at the onset of CHD; (2) have no symptoms of angina or possible MI by WHO questionnaire assessments [15]; (3) have no history of CHD diagnosed by a doctor; and (4) have a resting 12 lead ECG lead showing no evidence of ischaemia or previous MI [16].

All subjects underwent physical examination and provided demographic information and medical history (including CHD risk factors) using standardised questionnaires.

The study was approved by the Research Ethics Committee of Queen's University Belfast and informed consent was obtained from all subjects.

### DNA extraction and genotyping

DNA was extracted from peripheral whole blood using a salting out method. Genotype determination was performed using multiplex SNaPshot technology, an ABI fluorescence-based assay allelic discrimination method (Applied Biosystems, USA). Genotyping was repeated in 10% of the samples randomly selected as a quality control measure. Two observers unaware of the subject's disease status read each gel image. GeneMarkerV1.5 (Softgenetics, USA) was used to determine each allele.

### Statistical analysis

The combined TDT/S-TDT test [17,18] was used to assess the presence of linkage disequilibrium between the 6 SNPs or 5 haplotypes and early-onset CHD by testing for unequal transmission of an allele or haplotype from parents to affected offspring or unequal sharing of an allele or haplotype within disease-discordant sibships.

The combined TDT/S-TDT combines the TDT with the sibling TDT (S-TDT). Trios are informative for the TDT if there is an affected child and at least one parent is heterozygous. Sib pairs are informative for the S-TDT if there is at least one affected and one unaffected sibling with different marker genotypes.

Analysis of variance was used to compare means for quantitative variables and the chi-squared test was used for qualitative variables. SPSS 14.0 (SPSS Inc, USA) was used for these statistical analyses.

All statistical tests were performed at the 5% significance level (two-tailed).

## Results

A total of 1494 individuals from 580 families were included (800 discordant sib-pairs and 64 parent-child trios). Table 1 shows the family structures of the participants. Of note, there are more male probands and more female siblings, which reflect the earlier onset of CHD in men compared with women (Table 2). Smoking and diabetes are more common in the probands; however, hypertension and elevated levels of total cholesterol were less common in the probands, probably reflecting pharmacological intervention in the patient group (Table 2). TDT/S-TDT results at the single SNP level are shown in Table 3. No transmission distortion was detected for any of the tested SNPs. Table 4 shows the association analysis of CFH gene haplotypes. No association between any of the haplotypes and early-onset CHD was found.

**Table 1:** Family structures of 1494 subjects in 580 families

	Number of families	Number of individuals
Proband + parents + 0 sibling	48	144
Proband + parents + 1 sibling	9	36
Proband + parents + 2 sibling	7	35
Proband+1 sibling	337	674
Proband+2 sibling	128	384
Proband+3 sibling	39	156
Proband+4 sibling	8	40
Proband+5 sibling	3	18
Proband+6 sibling	1	7
Total	580	1494

**Table 2:** Risk factors in the probands and their siblings with premature onset CHD

Risk factor	Probands (n = 580)	Siblings (n = 786)
Age*	52.0(SD = 7.4)	56.0(SD = 7.8)
Female*	113 (19.5%)	429 (54.6%)
Male*	467 (80.5%)	357 (45.4%)
Non smoker*	116 (20.0%)	328 (41.7%)
Ex smoker*	249 (42.9%)	224 (28.5%)
Current smoker*	215 (37.1%)	234 (29.8%)
Hypertension treatment	148 (25.5%)	177 (22.5%)
Systolic BP ≥ 140 mmHg	30 (5.2%)	239 (30.4%)
Diastolic BP ≥ 95 mmHg	1 (0.2%)	2 (0.3%)
Total hypertension*	179 (30.9%)	418 (53.2%)
Known diabetes*	53 (9.1%)	43 (5.5%)
Total cholesterol (mmol/l)*	4.9(SD = 1.1)	5.8(SD = 1.1)

* P < 0.01, SD – standard deviation.

**Table 3:** Association tests between 6 SNPs and premature heart disease performed using the Transmission Disequilibrium Test/sibling Transmission Disequilibrium Test (TDT/S-TDT)

SNP name	Coding variant	Allele	Number of informative families	W^1^	Expected(W)	Variance(W)	P-value
rs 800292	I62V	CT	218	289	288.7	60.4	0.97
rs 1061170	Y402H	CT	323	300	294.9	107.0	0.62
rs 2274700	A473A	AG	203	132	128.2	57.0	0.62
rs 3753396	Q672Q	AG	218	314	310.7	61.1	0.67
rs 419137		AC	181	253	254.0	48.4	0.89
rs 2284664		AG	216	142	141.3	59.9	0.93

^1 ^W = X+Y where X is the number of transmissions of the first-mentioned allele from heterozygote parents to affected siblings (TDT) and Y is the number of occurrences of the first-mentioned allele in affected sibs in remaining informative families (S-TDT).

The 6 SNPs are arranged in an order according to their positions in the CFH gene.

**Table 4:** Association between 5 CFH haplotypes and premature heart disease

Haplotypes	Sequence	W^1^	Expected(W)	Variance(W)	P-value
H1	CCGACG	133	131.9	47.9	0.88
H2	CCGAAG	279	280.4	97.5	0.89
H3	CTGGAG	181	184.5	61.6	0.66
H4	TTGAAA	167	167.7	58.5	0.92
H5	CTAAAG	166	161.5	56.6	0.55

^1 ^W = X+Y where X is the number of transmissions of the haplotype from heterozygote parents to affected siblings (TDT) and Y is the number of the occurrences of the haplotype in affected sibs in remaining informative families (S-TDT).

The 6 alleles in the Sequence column from left to right are from rs800292 to rs2284664 according to their positions in the CFH gene.

## Discussion

There is evidence that polymorphisms in the CFH gene may be involved in atherogenesis [19]. The CFH gene is a member of the Regulator of Complement Activation (RCA) gene cluster [5]. The activation of the complement system is an important link between inflammation and atherogenesis. It has been postulated that variants in the CFH gene may be associated with CHD through modulation of inflammatory pathways, as has been reported in age-related macular degeneration (AMD) patients [4].

The CFH gene has been found to contribute around 50% percent of the risk of AMD [5]. AMD and atherosclerotic cardiovascular disease appear to share common pathogenic mechanisms: both are characterized by lipid deposition (drusen and plaques) and thickening of connective tissue (Bruch's membrane and arterial intima) [20]. One possible pathogenic mechanism is that the normal role of CFH is to prevent uncontrolled complement activation and inflammation [6]. Hence, mutations in CFH may increase inflammation and its pathological consequences. However, there is evidence that the mechanisms of atherogenesis may vary according to the size of arteries, which means that whilst CFH genetic variants may contribute to the atherogenesis in the tiny vessels in the eye, they may not have the same effect in the coronary arteries [21].

A recent study in Netherlands showed that the Y402H polymorphism was associated with increased risk of myocardial infarction. The HH homozygotes had a hazard ratio of 1.77 (95% confidence interval 1.23 to 2.55) [10]. They first excluded the non-MI CHD patients (13% of the study population). In their case-control study, using samples from the Physicians' Health Study, Zee et al did not find any association between the CFH Y402H gene polymorphism and incident myocardial infarction, ischaemic stroke, or venous thromboembolism [11]. Using samples from the Atherogene and ECTIM studies, Nicaud and colleagues were also unable to find any association between common polymorphisms in the CFH and coronary artery disease [12]. Likewise, in their study of 1170 patients with angiographically confirmed coronary artery disease and 560 controls, Goverdhan and co-workers did not detect any association between the CFH Y402H gene variant and presence or severity of CAD [13]. Such non-replication of findings is a common finding in the field of the genetics of complex disease [22].

One possible explanation for the lack of association in some of these reported studies is that CFH may be involved more in the formation of unstable plaques rather than the more stable atherosclerotic lesions. A significant characteristic of unstable lesions is the presence of numerous macrophages with matrix metalloproteinases, which can digest the fibrous cap part of the plaque. In the paper by Sivaprasad and colleagues, 3-fold increases of matrix metalloproteinase-9 were found in the AMD patients [23]. This function may be mediated in part by C-Reactive Protein, which has been reported to be associated with unstable atherosclerotic lesions [24]. Another supporting study showed that the activation of CFH is greater in the superficial rather than the deeper layer of the atherosclerotic plaque [2].

Whilst we have shown differences in the prevalence of conventional risk factors between the probands and siblings, family-based statistical analyses are, unfortunately, not suited to investigating gene-environment interactions since, unlike the subjects in a case-control study, families cannot be neatly divided into those with and those without the environmental risk factor of interest (such as smoking status or diabetes).

Although free from the problems of hidden population stratification, there is a relative lack of power with family based association studies. The number of families recruited was 580; however, the number of informative families ranged from 181 to 323 (Table 3). Of course, we cannot exclude the possibility that other polymorphisms in the CFH gene, which are not in linkage disequilibrium with any of these 6 SNPs, may play a role. However, we found no association between 6 common SNPs or 5 haplotypes of the CFH gene and early-onset CHD in this Irish family-based population.

## Conclusion

In this family-based study, we found no evidence of an association between variants of the CFH gene and early-onset coronary heart disease.

## Competing interests

The author(s) declare that they have no competing interests.

## Authors' contributions

WM undertook the experimental work and wrote the manuscript; AH supervised the study; CCP performed statistical analysis; CB provided technical support; MSK and PGH collected patient samples and helped to draft the manuscript. FK and PPM provided overall direction to the project and revised the manuscript. All authors have read and approved the final manuscript.

## Pre-publication history

The pre-publication history for this paper can be accessed here:


